# Chronic Kidney Disease and Infection Risk: A Lower Incidence of Peritonsillar Abscesses in Specific CKD Subgroups in a 16-Year Korean Nationwide Cohort Study

**DOI:** 10.3390/microorganisms12122614

**Published:** 2024-12-18

**Authors:** Mi Jung Kwon, Ho Suk Kang, Joo-Hee Kim, Ji Hee Kim, Woo Jin Bang, Dae Myoung Yoo, Na-Eun Lee, Kyeong Min Han, Nan Young Kim, Hyo Geun Choi, Min-Jeong Kim, Eun Soo Kim

**Affiliations:** 1 Department of Pathology, Hallym University Sacred Heart Hospital, Hallym University College of Medicine, Anyang 14068, Republic of Korea; mulank99@hallym.or.kr; 2 Division of Gastroenterology, Department of Internal Medicine, Hallym University Sacred Heart Hospital, Hallym University College of Medicine, Anyang 14068, Republic of Korea; hskang76@hallym.or.kr; 3 Division of Pulmonary, Allergy, and Critical Care Medicine, Department of Medicine, Hallym University Sacred Heart Hospital, Hallym University College of Medicine, Anyang 14068, Republic of Korea; luxjhee@gmail.com; 4 Department of Neurosurgery, Hallym University Sacred Heart Hospital, Hallym University College of Medicine, Anyang 14068, Republic of Korea; kimjihee.ns@gmail.com; 5 Department of Urology, Hallym University Sacred Heart Hospital, Hallym University College of Medicine, Anyang 14068, Republic of Korea; yybbang@hallym.or.kr; 6 Hallym Data Science Laboratory, Hallym University College of Medicine, Anyang 14068, Republic of Korea; ydm@hallym.ac.kr (D.M.Y.); intriguingly@hallym.ac.kr (N.-E.L.); km.han@hallym.ac.kr (K.M.H.); 7 Laboratory of Brain and Cognitive Sciences for Convergence Medicine, Hallym University College of Medicine, Anyang 14068, Republic of Korea; 8 Hallym Institute of Translational Genomics and Bioinformatics, Hallym University Medical Center, Anyang 14068, Republic of Korea; honeyny@hallym.or.kr; 9 Suseo Seoul E.N.T. Clinic, 10, Bamgogae-ro 1-gil, Gangnam-gu, Seoul 06349, Republic of Korea; mdanalytics@naver.com; 10 Department of Radiology, Hallym University Sacred Heart Hospital, Hallym University College of Medicine, Anyang 14068, Republic of Korea; drkmj@hallym.or.kr

**Keywords:** chronic kidney disease, peritonsillar abscess, deep neck infection, risk factor, public health, longitudinal cohort study, big data analysis

## Abstract

Peritonsillar abscesses and deep neck infection are potentially serious infections among patients with chronic kidney disease (CKD), posing risks for severe complications and drawing significant public health concern. This nationwide, population-based longitudinal study (2002–2019) assessed the extended relationship between chronic kidney disease (CKD) and the likelihood of peritonsillar abscess and deep neck infection in a Korean cohort. Using a 1:4 propensity score overlap-weighted matching, we included 16,879 individuals with CKD and 67,516 comparable controls, accounting for demographic variables and comorbidities to ensure balanced group comparisons. Hazard ratios (HRs) and 95% confidence intervals (CIs) for deep neck infection and peritonsillar abscesses in relation to CKD history were calculated with a propensity score overlap-weighted Cox proportional hazards model. Our results revealed no significant increase in the overall incidence of deep neck infections or peritonsillar abscesses in CKD patients compared to controls. Interestingly, CKD patients demonstrated a 50% reduced likelihood of developing peritonsillar abscesses (HR 0.50; 95% CI = 0.30–0.83; *p* = 0.007), particularly among subgroups aged 70 years or older, females, non-smokers, rural residents, overweight individuals, and those with lower comorbidity burdens (e.g., absence of hypertension, hyperlipidemia, or hyperglycemia). In summary, the results suggest that lifestyle modifications and the effective management of comorbidities could reduce the risk of peritonsillar abscess in certain CKD subgroups. Our findings may help to alleviate public health concerns regarding peritonsillar abscesses and deep neck infections as CKD-related comorbidities.

## 1. Introduction

Chronic kidney disease (CKD), defined by ongoing impairments in kidney function or structure lasting over three months [1], is a global health concern, impacting an estimated 10–13% of the population in South Korea [2,3]. CKD patients are particularly vulnerable to infections due to immune system dysfunction and a high burden of comorbidities, including diabetes, cardiovascular disease, and hypertension [4]. Infections are a significant cause of morbidity and hospitalizations in CKD patients, accounting for up to 21% of all admissions in this population [4]. Among the infections affecting CKD patients, deep neck infections and peritonsillar abscesses warrant special attention due to their severity and significant impact on clinical outcomes, including risks of airway obstruction, sepsis, and even fatality if left untreated [5,6].

A peritonsillar abscess, a localized infection near the tonsils, is characterized by pus accumulation between the palatine tonsillar capsule and the superior pharyngeal constrictor muscle [7]. Severe peritonsillar abscesses can extend to surrounding areas, potentially leading to deeper neck infections that demand urgent medical intervention [5]. Prompt drainage, microbial culture, intravenous antibiotics, and sometimes surgical procedures like tracheostomy are necessary for managing deep neck infections to prevent life-threatening complications [5].

Recent studies suggest a possible link between CKD and an increased risk of these serious infections, with some evidence indicating higher rates of peritonsillar abscess and deep neck infections in end-stage renal disease patients [8,9]. These two cohort studies indicate that advanced kidney disease may be a predisposing factor for both deep neck infections [9] and peritonsillar abscesses [8], with higher mortality observed in affected CKD patients [9]. This association highlights potential concerns regarding the elevated comorbidity risk of these infections in individuals with CKD. Other research has also suggested that microbial interactions within the tonsil, particularly with the tonsillar microbiota, may contribute to the pathophysiology of kidney diseases, potentially linking oral health to systemic inflammation in CKD patients [10]. For example, *Tannerella* species, common in the oral cavity and associated with periodontitis, have been linked to CKD [11]. The immunological impact of CKD may explain this infection risk [12]. Persistent renal injury and decreased renal function are associated with chronic inflammation, oxidative stress, and the accumulation of uremic toxins that impair immune cell function, including neutrophils and macrophages [13,14]. This immune dysregulation may increase susceptibility to infections [12], including deep neck infections and peritonsillar abscesses.

However, despite the importance of understanding these risks, there have been few large-scale epidemiological studies to validate the relationship between CKD and deep neck infections or peritonsillar abscesses [8,9]. Existing studies have several limitations: they lack balanced sample sizes between CKD and control groups and do not account for a wide range of comorbidities [8,9]. In addition, these studies employed 1:1 matching [8,9], which can lead to significant data loss when propensity scores vary between groups, potentially resulting in selection bias and underrepresenting certain patient characteristics [15]. To accurately assess the effect of CKD on the risk of experiencing deep neck infections and peritonsillar abscesses, further research using well-matched, large-scale cohort data that adjusts for confounding variables is essential. Since CKD and these infections may have overlapping risk factors and potential bidirectional links, a carefully controlled longitudinal study is needed to investigate these relationships comprehensively.

In this study, we hypothesized a potential positive association between CKD and the risk of developing deep neck infection or a peritonsillar abscess, which may vary based on individual patient characteristics, such as age, sex, socioeconomic status, and comorbid conditions. The primary aim was to explore the relationship between CKD and the risk of peritonsillar abscess and deep neck infections. Additionally, we examined whether certain lifestyle or demographic factors (e.g., age, sex, smoking, rural residence) might influence this risk within the CKD population. To fulfill this, we conducted a long-term observational study using data from the Korean national public healthcare system, analyzing the association between CKD and infection risk while accounting for potential confounding factors. This research could help to inform more targeted infection prevention strategies and improve clinical outcomes for CKD patients, addressing an important public health concern.

## 2. Materials and Methods

### 2.1. Data Resource

This study draws on a robust resource, the Korean National Health Insurance Service-Health Screening Cohort (KNHIS-HSC) database, which is designed to support academic research through anonymized, population-based records, as previously documented [16]. Established in 1999, the Korean National Health Insurance Service (KNHIS) provides obligatory medical coverage to around 97% of the Korean people, while the remaining 3% are supported through medical aid programs. The KNHIS-HSC cohort includes individuals who participated in health screenings in 2002 and 2003, aged 40 to 79 at the time of enrollment, with follow-up extending through 2019 [17]. The cohort comprises 514,866 participants, selected via a 10% simple random sampling from all eligible individuals during these years [17]. All data in the KNHIS-HSC are anonymized with scrambled identification codes, and diagnoses are coded according to the International Classification of Diseases, 10th Revision, Clinical Modification (ICD-10-CM). For additional details on the structure of the KNHIS-HSC database, refer to prior publications [17,18,19]. This study was authorized by the Ethics Committee of Hallym University (IRB No. 2022-10-008), with a waiver of informed consent in compliance with all relevant ethical guidelines.

### 2.2. Exposure (Chronic Kidney Disease) and Outcome (Deep Neck Infection and Peritonsillar Abscess)

Participants with CKD were identified by having at least two diagnoses of CKD (ICD-10 code N18) or unspecified kidney failure (N19). Additionally, individuals receiving routine dialysis, such as hemodialysis or peritoneal dialysis, were incorporated if they had the following therapeutic codes: O7010, O7020, and O7070 [20].

Deep neck infection was identified using ICD-10 codes J39.0 and J39.1 and included only participants who required surgical intervention, as indicated by claim codes Q2250, Q2251, and Q2252 [21]. A peritonsillar abscess was identified with ICD-10 code J36, with inclusion limited to cases treated by incision, drainage, or aspiration (claim code Q2320) [21].

### 2.3. Cohort Selection

**Figure 1:**
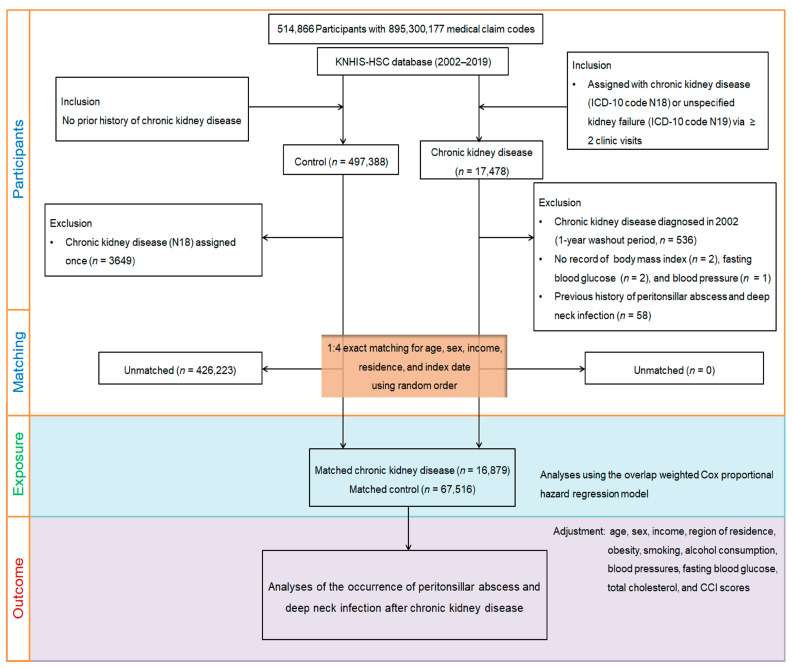
A visual overview of the participant selection and matching process in the study. Starting with the initial pool of 514,866 individuals in the Korean National Health Insurance Service-Health Screening Cohort (KNHIS-HSC) database, a meticulous selection process resulted in 16,879 patients diagnosed with chronic kidney disease being matched with 67,516 control participants. Matching was based on age, sex, income, and region of residence.

We retrospectively designed a time-based cohort study to assess the effect of CKD on the incidence of deep neck infection and peritonsillar abscess, comparing a CKD cohort with a non-CKD control group. CKD cases were identified using the inclusion criteria described above, selecting from a pool of 514,866 participants aged ≥40 with 895,300,177 medical claims from at least two clinic visits between 2002 and 2019 (*n* = 17,478) (Figure 1). The control group consisted of participants without a history of CKD diagnosis (*n* = 497,388), excluding individuals with a single ICD-10 code N18 (CKD) assignment (*n* = 3649). To ensure only new CKD diagnoses were included, we excluded participants diagnosed in 2002 (1-year wash-out period, *n* = 536) and those lacking documentation of their blood pressure (*n* = 1), fasting blood glucose (*n* = 2), or body mass index (BMI) (*n* = 2). Individuals with a history of deep neck infection or peritonsillar abscesses prior to the index date were also excluded (*n* = 58).

Propensity score matching was conducted at a 1:4 ratio based on sex, age, income level, and residential region to minimize baseline demographic and clinical differences between groups and reduce potential confounding. This ratio was chosen based on evidence suggesting that larger ratios provide limited additional statistical benefit [22]. Non-CKD controls were randomly selected, and the index date for each CKD participant was set as the date of CKD diagnosis. For matched controls, the index date corresponded to the CKD participant’s index date. To ensure a balanced cohort, controls who had died or developed a deep neck infection or peritonsillar abscess prior to the index date were excluded, resulting in the removal of 426,223 control participants. No CKD cases were unmatched during this process. Although this exclusion led to a reduction in the number of controls, it allowed for the creation of a well-matched and comparable cohort for analysis [22]. Ultimately, 16,879 CKD patients were matched to 67,516 controls using the 1:4 matching ratio. New cases of deep neck infection and peritonsillar abscess were tracked in both groups using ICD-10 codes from each participant’s index date until 31 December 2019.

### 2.4. Variables

Participants were grouped into ten age categories (each spanning five years) and five income levels, from the lowest to the highest, allowing for detailed socioeconomic analysis. Residential areas were divided into urban (the seven largest cities) and rural regions based on population size and administrative boundaries [23]. Lifestyle factors included smoking status, alcohol drinking, and weight status as measured by BMI [24]. Physiological measures of blood pressure, fasting blood glucose, and total cholesterol were recorded for a comprehensive health profile.

To evaluate overall disease burden, the Charlson Comorbidity Index (CCI) was employed, assigning each participant a score on the basis of the presence and severity of 17 diseases. This score [0 (no accompanying illnesses)–29 (numerous serious comorbidities)] provided a standardized method to assess cumulative health impact across participants [25].

### 2.5. Statistics

The standardized difference was exerted to juxtapose baseline characteristics between cohorts. Propensity score overlap weighting was applied to achieve covariate balance, maximize the effective sample size, and reduce intergroup bias. CKD participants were weighted based on the propensity score, while control participants were weighted by the complement of the propensity score (1 minus the propensity score). This overlap weighting approach, scaled from 0 to 1, ensures precise balance and enhances analytical accuracy [26,27]. Absolute standardized differences for covariates before and after matching were assessed, with values ≤ 0.20 indicating adequate balance [28].

Crude incidence rates (IR) and incidence rate differences (IRD) were calculated by dividing the number of events by person-years and expressed as cases per 1000 person-years. We used the Kaplan–Meier estimator to estimate the probability of deep neck infection and peritonsillar abscess occurrence over time among participants still at risk. A log-rank test was applied to determine statistically significant differences in survival rates between the CKD and control groups. We employed overlap weighting based on propensity scores to balance baseline covariates (e.g., age, sex, income, region of residence, and clinical factors) between groups. This method mimics the properties of a randomized trial and provides a more accurate estimation of the relationship between CKD and infection risk [27]. Hazard ratios (HRs) and 95% confidence intervals (CIs) for deep neck infection and peritonsillar abscesses in relation to CKD history were estimated using a propensity score overlap-weighted Cox proportional hazards regression model. All unadjusted models and overlap-weighted adjusted models were analyzed. Proportional hazard assumptions were verified using log-minus-log plots, with no violations observed. Subgroup analyses were performed for all covariates. Statistical analyses were conducted using two-tailed tests, with *p*-values < 0.05 considered statistically significant. Statistics in this study were operated running SAS 9.4 software (SAS Institute Inc., Cary, NC, USA).

## 3. Results

### 3.1. Cohort Features

The cohort included 16,879 CKD individuals and a control group of 67,516 people. In the unadjusted analysis, the matching process resulted in four covariates—age, sex, income, and residence—having a standardized difference of 0.00, showing no initial differences between two groups for these variables. However, imbalances were noted in other baseline characteristics, such as fasting blood glucose levels and CCI scores, with standardized differences exceeding 0.2. After applying overlap weighting adjustments, these imbalances were either eliminated (standardized difference = 0.00) or substantially reduced (standardized difference ≤ 0.2), achieving a well-aligned distribution of demographic and clinical characteristics between the CKD and control groups (Table 1). All analyses were conducted on this matched cohort, derived using a 1:4 propensity score matching ratio to balance baseline characteristics and minimize potential confounding.

In the unadjusted analysis, the matching process resulted in four covariates—age, sex, income, and residence—having a standardized difference of 0.00, indicating no initial differences between the CKD and control groups for these variables. However, imbalances were noted in other baseline characteristics, such as fasting blood glucose levels and CCI scores, with standardized differences exceeding 0.2. After applying overlap weighting adjustments, these imbalances were either eliminated (standardized difference = 0.00) or substantially reduced (standardized difference ≤ 0.2), achieving a well-aligned distribution of demographic and clinical characteristics between the CKD and control groups.

### 3.2. Incidence of Deep Neck Infection in the CKD and Control Groups

The crude and adjusted HRs for deep neck infection in the CKD cohort were not markedly different from those in the comparison group ([HR, 1.56; 95% CI = 0.50–4.84; *p* = 0.442] and [HR 1.76; 95% CI = 0.68–4.56; *p* = 0.242], in that order) (Table 2). During the follow-up period, deep neck infections occurred in 4 patients with CKD (0.02%) and 12 controls (0.02%). Kaplan–Meier analysis revealed an absence of notable difference in probability of a deep neck infection incidence between the CKD and comparison groups (*p* = 0.4380).

Similarly, subgroup analyses across all covariates revealed no significant associations between CKD and the likelihood of deep neck infection compared to controls (Supplementary Table S1).

### 3.3. Incidence of Peritonsillar Abscess in the CKD and Control Groups

Peritonsillar abscess was observed in 9 participants (0.05%) in the CKD group (*n* = 16,879) and 79 participants (0.12%) in the control group (*n* = 67,516). Over the follow-up period, the cohort generated 71,876 person-years in the CKD group and 350,135 person-years in the control group, resulting in incidence rates of peritonsillar abscess of 0.13 and 0.23 per 1000 person-years, in that order.

Although the Kaplan–Meier method and log-rank test suggested a lower probability of incidence of peritonsillar abscess among CKD participants compared to non-CKD participants, the result did not reach statistical significance (*p* = 0.0745). In the unadjusted model, the crude hazard ratio (HR) for peritonsillar abscess incidence also showed no significant difference among those with CKD and the control subjects (HR 0.54; 95% CI = 0.27–1.07; *p* = 0.079). However, after adjusting for potential confounding variables using overlap weighting, CKD patients were found to have a significantly reduced likelihood of developing peritonsillar abscess (HR 0.50; 95% CI = 0.30–0.83; *p* = 0.007) (Table 3).

Detailed subgroup analyses further supported this association, with the reduced risk of peritonsillar abscess in CKD patients persisting significantly among those who were aged 70 years or older, females, non-smokers, rural residents, overweight individuals, those who drank alcohol less than or more than once a week, those with a CCI score ≥ 2, and those without hyperglycemia, hyperlipidemia, or hypertension (Supplementary Table S2).

## 4. Discussion

Using an overlap-weighted Cox regression model that accounted for comorbidities, demographic factors, and socioeconomic characteristics, our results showed no significant increase in the overall incidence of deep neck infections or peritonsillar abscesses among CKD patients compared to controls over the 16-year follow-up period. Interestingly, however, CKD patients exhibited a 50% reduction (95% CI = 0.30–0.83; *p* = 0.007) in the likelihood of peritonsillar abscess, despite the Kaplan–Meier method and unadjusted model showing no statistically significant differences. This finding underscores the importance of accounting for baseline imbalances, such as differences in fasting blood glucose, comorbidity burden, and other variables that may influence infection risk. The overlap-weighted model provided a more precise estimate of the association by accounting for imbalances in baseline covariates, mimicking conditions of a randomized trial [27]. This highlights the need for caution when interpreting crude analyses that do not adjust for potential confounders.

The reduced likelihood of developing peritonsillar abscesses in CKD patients was particularly notable among elderly females, rural residents, non-smokers, overweight individuals, and those with fewer comorbidities, such as the absence of hypertension, hyperlipidemia, or hyperglycemia. For elderly females and non-smokers, this reduced risk may be linked to a lower prevalence of known risk factors, including smoking, recurrent tonsillitis, bacterial infections (e.g., Group A *Streptococcus* and *Staphylococcus aureus*), poor oral hygiene, immunocompromised status, a younger age, and male sex [29,30] within these subgroups. For rural residents, overweight individuals, and those without hypertension, hyperlipidemia, or hyperglycemia, the reduced risk may be attributed to lifestyle and demographic factors, such as reduced exposure to urban pollution and healthier weight profiles in these populations, because factors such as low BMI and the presence of diabetes or cardiovascular disease were associated with a higher infection risk [31], and malnutrition accelerates the occurrence of infectious complications in patients with CKD [32]. Overall, our findings may highlight that lifestyle modifications and the effective management of comorbidities can help protect against infections in specific CKD subgroups. These results may emphasize the potential benefits of individualized risk assessments, particularly for older female CKD patients without significant metabolic or lifestyle risk factors.

Our findings appear to contrast with previous studies reporting a higher overall risk of infections in CKD patients [31,32]. For instance, one study noted an increased incidence of community-acquired infections in mild to severe CKD but found no significant association with nervous system or upper respiratory tract infections, suggesting variability in site-specific infection risks [33]. Given the close link between peritonsillar abscess, deep neck infections, and upper respiratory tract infections [6,11], this variability may explain the differing results.

Limited research has explored the relationship between CKD and peritonsillar abscesses or deep neck infections, with only two Taiwanese studies addressing this context [8,9]. These studies, focused on end-stage renal disease, reported a 1.98-fold increased risk of peritonsillar abscess [8] and a 2.23-fold higher risk of deep neck infections [9]. In contrast, our study, which included patients across all CKD stages, found a 50% reduced likelihood of peritonsillar abscess and no significant association with deep neck infections. The higher infection rates in end-stage renal disease patients, whose risk is 4–10 times greater than in mild CKD [12], likely contribute to these differences. Additionally, variations in CKD prevalence, patient characteristics, and healthcare systems between Taiwan and Korea may further explain the contrasting findings. For example, Taiwan has higher rates of hypertension, obesity (BMI > 30 kg/m^2^), and renal replacement therapy prevalence [34,35], ranking first globally in end-stage renal disease incidence [35].

Methodological differences also play a role. Unlike the 1:1 propensity score matching in the Taiwanese studies [8,9], we used a 1:4 matching ratio, balancing 16,879 CKD patients with 67,516 controls to minimize data loss and improve statistical power [22]. Our rigorous design adjusted for a wide range of confounders, including demographic, socioeconomic, lifestyle, and comorbidity factors, providing a more comprehensive analysis. These adjustments allowed us to observe a potential reduction in peritonsillar abscess risk among CKD patients, with no significant association with deep neck infections, alleviating some public health concerns regarding these conditions as CKD-related comorbidities.

The mechanisms linking CKD to a potentially reduced risk of peritonsillar abscess are not yet fully understood, though several factors may contribute. Uremia and its treatment can alter the biochemical environment of various organs—including the heart, lungs, liver, brain, and gut—through the influence of inflammatory cytokines and immune cells [14]. CKD is known to induce immunological changes, such as modified T-cell responses and chronic low-grade inflammation, which may affect inflammatory pathways and reduce susceptibility to certain bacterial infections, including peritonsillar abscess [36]. For instance, the uremic environment in CKD is associated with altered helper T-cell responses, possibly explaining the reduced antibody responses observed in these patients [36]. Consistent with this, studies have shown that patients undergoing hemodialysis produce fewer antigen-specific memory CD4+ T cells following vaccination [37].

In addition, CKD disrupts tryptophan catabolism, resulting in elevated levels of indoxyl sulfate and other metabolites due to gut dysbiosis. This disruption leads to an increased prevalence of phenol- and p-cresol-producing bacteria (e.g., *Enterobacteriaceae*, *Enterococcaceae*, and *Clostridium perfringens*) in the gut microbiome, which may influence microbial communities in the oral and pharyngeal regions, potentially affecting pathogen colonization [38,39]. Since the tonsils have a key function in antigen processing within the mucosal immune system and serve as the first defense against pathogens entering through the gastrointestinal tract [10], CKD-associated changes in the gut microbiome may extend to the oral and pharyngeal microbiomes. This shift could alter pathogen colonization patterns and potentially reduce susceptibility to specific infections, including peritonsillar abscesses [38,39].

This study’s robustness and credibility were enhanced by using a verified national cohort dataset, which allowed for well-matched patient and comparison groups via overlap-weighted propensity score matching, reducing selection bias and enabling subgroup analyses akin to randomized clinical trials [27]. Access to comprehensive medical histories through the KNHIS-HSC database improved the generalizability and precision of our findings, further strengthened by adjustments for potential confounders, including socioeconomic, lifestyle, and comorbidity factors. Our 16-year observational period, one of the most extended to explore the link between CKD and infection risk, yielded a unique advantage in identifying long-term associations.

However, certain limitations should be acknowledged. As an observational retrospective study, causal relationships between CKD and infection risk cannot be definitively established and we did not investigate the root causes that might explain those correlations. Focusing on Korean adults over 40 and relying on administrative data (e.g., ICD-10 codes) may limit the generalizability of our findings and introduce potential inaccuracies due to misclassification or the underreporting of diagnoses. The KNHIS-HSC database lacks detailed information on CKD severity (e.g., estimated glomerular filtration rate, proteinuria), medication history (e.g., vaccinations, antibiotics), genetic factors, and specific clinical markers, such as creatinine levels. These data gaps hinder a more comprehensive analysis of how CKD severity and individual factors influence the risk of peritonsillar abscesses and deep neck infections. Another limitation is the small number of events in the study, with only 16 deep neck infections and 88 peritonsillar abscesses observed. This limited event count may increase the risk of over-analysis and reduce the generalizability of our findings. Addressing these residual confounders and including a larger sample with more events in future studies will be essential to validate and refine these results.

## 5. Conclusions

This large, nationwide, population-based study may suggest that the overall CKD population in Korea may not face an overall increased risk for peritonsillar abscesses or deep neck infection compared to individuals without CKD. Notably, CKD patients exhibited a 50% lower likelihood of developing a peritonsillar abscess than controls, particularly among certain subgroups such as older females, rural residents, non-smokers, those who are overweight, and those with fewer comorbidities (e.g., without hypertension, hyperlipidemia, or hyperglycemia). These findings underscore that not all CKD patients face uniformly higher infection risks and highlight the importance of lifestyle modifications and effective comorbidity management. Health professionals should consider individualized risk assessments, especially for older female CKD patients without significant metabolic or lifestyle risk factors, to optimize care and reduce infection risks.

## Figures and Tables

**Table 1:** Baseline demographic and clinical attributes of participants before and after overlap weighting adjustments.

Characteristics	Before Overlap Weighting Adjustment			After Overlap Weighting Adjustment		
	CKD	Control	Standardized Difference	CKD	Control	Standardized Difference
Age (years; n, %)			0.00			0.00
40–44	98 (0.58)	392 (0.58)		73 (0.61)	73 (0.61)	
45–49	363 (2.15)	1452 (2.15)		250 (2.10)	250 (2.10)	
50–54	947 (5.61)	3788 (5.61)		654 (5.51)	654 (5.51)	
55–59	1859 (11.01)	7436 (11.01)		1293 (10.90)	1293 (10.89)	
60–64	2298 (13.61)	9192 (13.61)		1598 (13.47)	1598 (13.47)	
65–69	2606 (15.44)	10,424 (15.44)		1807 (15.23)	1807 (15.23)	
70–74	2970 (17.60)	11,880 (17.60)		2091 (17.62)	2091 (17.62)	
75–79	2898 (17.17)	11,592 (17.17)		2062 (17.38)	2063 (17.38)	
80–84	1908 (11.30)	7632 (11.30)		1360 (11.46)	1360 (11.46)	
85+	932 (5.52)	3728 (5.52)		676 (5.70)	676 (5.70)	
Sex (n, %)			0.00			
Male	11,087 (65.69)	44,348 (65.69)		7821 (65.93)	7821 (65.93)	
Female	5792 (34.31)	23,168 (34.31)		4042 (34.07)	4042 (34.07)	
Income (n, %)			0.00			0.00
1 (lowest)	2948 (17.47)	11,792 (17.47)		2047 (17.26)	2047 (17.26)	
2	1942 (11.51)	7768 (11.51)		1358 (11.45)	1358 (11.45)	
3	2404 (14.24)	9616 (14.24)		1680 (14.16)	1680 (14.16)	
4	3349 (19.84)	13,396 (19.84)		2351 (19.82)	2351 (19.82)	
5 (highest)	6236 (36.95)	24,944 (36.95)		4427 (37.32)	4427 (37.32)	
Region of residence (n, %)			0.00			0.00
Urban	7238 (42.88)	28,952 (42.88)		5092 (42.92)	5092 (42.92)	
Rural	9641 (57.12)	38,564 (57.12)		6771 (57.08)	6771 (57.08)	
Obesity ^†^ (n, %)			0.16			0.00
Underweight	446 (2.64)	2320 (3.44)		333 (2.81)	333 (2.81)	
Normal	5173 (30.65)	24,073 (35.66)		3753 (31.63)	3753 (31.63)	
Overweight	4428 (26.23)	18,259 (27.04)		3149 (26.54)	3149 (26.54)	
Obese I	6066 (35.94)	20,965 (31.05)		4158 (35.05)	4158 (35.05)	
Obese II	766 (4.54)	1899 (2.81)		471 (3.97)	471 (3.97)	
Smoking status (n, %)			0.02			0.00
Non-smoker	10,793 (63.94)	43,640 (64.64)		7605 (64.10)	7605 (64.10)	
Past smoker	1770 (10.49)	7119 (10.54)		1259 (10.61)	1259 (10.61)	
Current smoker	4316 (25.57)	16,757 (24.82)		3000 (25.28)	3000 (25.28)	
Alcohol consumption (n, %)			0.07			0.00
<1 time a week	12,274 (72.72)	46,881 (69.44)		8512 (71.75)	8513 (71.75)	
≥1 time a week	4605 (27.28)	20,635 (30.56)		3351 (28.25)	3351 (28.25)	
SBP (mmHg; mean, SD)	131.81 (18.38)	128.69 (16.36)	0.18	130.68 (14.88)	130.68 (7.16)	0.00
DBP (mmHg; mean, SD)	78.73 (11.52)	78.08 (10.35)	0.07	78.45 (9.50)	78.45 (4.46)	0.00
FBG (mg/dL; mean, SD)	115.61 (49.22)	103.63 (28.27)	0.30	109.51 (31.43)	109.51 (16.44)	0.00
TG (mg/dL; mean, SD)	190.22 (45.65)	193.34 (39.18)	0.07	190.49 (37.69)	190.49 (16.61)	0.00
CCI score (mean, SD)	2.75 (2.52)	1.14 (1.74)	0.74	2.13 (1.78)	2.13 (1.02)	0.00
Peritonsillar abscess (n, %)	9 (0.05)	79 (0.12)	0.02	6 (0.05)	14 (0.12)	0.02
Deep neck infection (n, %)	4 (0.02)	12 (0.02)	0.00	3 (0.02)	2 (0.02)	0.01

Abbreviations: CKD, chronic kidney disease; CCI, Charlson Comorbidity Index; SBP, systolic blood pressure; SD, standard deviation; DBP, diastolic blood pressure; FBG, fasting blood glucose; TG, total cholesterol. ^†^ Obesity (BMI, body mass index, kg/m^2^) was categorized as <18.5 (underweight), ≥18.5 to <23 (normal), ≥23 to <25 (overweight), ≥25 to <30 (obese I), and ≥30 (obese II).

**Table 2:** Crude and overlap propensity score weighted hazard ratios (95% confidence interval) of CKD for deep neck infection with subgroup analyses according to age, sex, income, and region of residence.

	N of Event/N of Total (%)	Follow-Up Duration (PY)	IR Per 1000 (PY)	IRD (95% CI)	Hazard Ratios for Deep Neck Infection			
					Crude	*p*	Overlap-Weighted Model ^†^	*p*
Total participants								
CKD	4/16,879 (0.02)	71,911	0.06	0.03 (−0.03–0.07)	1.56 (0.50–4.84)	0.442	1.76 (0.68–4.56)	0.242
Control	12/67,516 (0.02)	350,496	0.03		1		1	
Age < 70 years old								
CKD	2/8171 (0.02)	46,196	0.04	0.02 (−0.02–0.07)	2.30 (0.42–12.5)	0.338	5.14 (0.84–31.4)	0.076
Control	4/32,684 (0.01)	218,139	0.02		1		1	
Age ≥ 70 years old								
CKD	2/8708 (0.02)	25,715	0.08	0.02 (−0.09–0.12)	1.22 (0.26–5.77)	0.799	1.14 (0.34–3.83)	0.827
Control	8/34,832 (0.02)	132,357	0.06		1		1	
Male								
CKD	3/11,087 (0.03)	46,207	0.06	0.03 (−0.02–0.10)	2.41 (0.60–9.64)	0.214	2.92 (0.86–9.99)	0.087
Control	6/44,348 (0.01)	225,084	0.03		1		1	
Female								
CKD	1/5792 (0.02)	25,704	0.04	−0.01 (−0.10–0.08)	0.75 (0.09–6.27)	0.794	0.58 (0.11–3.13)	0.524
Control	6/23,168 (0.03)	125,412	0.05		1		1	
Low income group								
CKD	3/7294 (0.04)	30,681	0.10	0.06 (−0.03–0.14)	2.36 (0.59–9.47)	0.224	2.95 (0.85–10.2)	0.088
Control	6/29,176 (0.02)	152,546	0.04		1		1	
High income group								
CKD	1/9585 (0.01)	41,230	0.02	−0.01 (−0.06–0.05)	0.77 (0.09–6.43)	0.812	0.70 (0.13–3.78)	0.678
Control	6/38,340 (0.02)	197,950	0.03		1		1	
Urban resident								
CKD	1/7238 (0.01)	32,775	0.03	0.00 (−0.06–0.07)	1.16 (0.13–10.4)	0.896	1.26 (0.25–6.38)	0.781
Control	4/28,952 (0.01)	156,595	0.03		1		1	
Rural resident								
CKD	3/9641 (0.03)	39,136	0.08	0.04 (−0.04–0.11)	1.77 (0.47–6.70)	0.398	2.12 (0.65–6.94)	0.212
Control	8/38,564 (0.02)	193,901	0.04		1		1	

Abbreviation: CKD, chronic kidney disease; IR, incidence rate; IRD, incidence rate difference; PY, person-year. Significance at *p* < 0.05. ^†^ Adjusted for age, sex, income, region of residence, obesity, smoking, alcohol consumption, systolic blood pressure, diastolic blood pressure, fasting blood glucose, total cholesterol, and CCI scores.

**Table 3:** Crude and overlap propensity score weighted hazard ratios (95% confidence interval) of CKD for peritonsillar abscess with subgroup analyses according to age, sex, income, and region of residence.

	N of Event/N of Total (%)	Follow-Up Duration (PY)	IR Per 1000 (PY)	IRD (95% CI)	Hazard Ratios for Peritonsillar Abscess			
					Crude	*p*	Overlap-Weighted Model ^†^	*p*
Total participants								
CKD	9/16,879 (0.05)	71,876	0.13	−0.10 (−0.22–0.02)	0.54 (0.27–1.07)	0.079	0.50 (0.30–0.83)	0.007 *
Control	79/67,516 (0.12)	350,135	0.23		1		1	
Age < 70 years old								
CKD	7/8171 (0.09)	46,161	0.15	−0.11 (−0.26–0.05)	0.57 (0.26–1.26)	0.165	0.57 (0.32–1.01)	0.053
Control	56/32,684 (0.17)	217,845	0.26		1		1	
Age ≥ 70 years old								
CKD	2/8708 (0.02)	25,715	0.08	−0.09 (−0.26–0.07)	0.43 (0.10–1.82)	0.251	0.28 (0.08–0.91)	0.034 *
Control	23/34,832 (0.07)	132,290	0.17		1		1	
Male								
CKD	6/11,087 (0.05)	46,184	0.13	−0.11 (−0.26–0.04)	0.51 (0.22–1.19)	0.119	0.59 (0.32–1.07)	0.080
Control	55/44,348 (0.12)	224,840	0.24		1		1	
Female								
CKD	3/5792 (0.05)	25,692	0.12	−0.07 (−0.25–0.10)	0.60 (0.18–2.00)	0.410	0.32 (0.12–0.90)	0.030 *
Control	24/23,168 (0.10)	125,295	0.19		1		1	
Low income group								
CKD	4/7294 (0.05)	30,674	0.13	−0.13 (−0.32–0.06)	0.48 (0.17–1.35)	0.164	0.51 (0.24–1.10)	0.085
Control	40/29,176 (0.14)	152,349	0.26		1		1	
High income group								
CKD	5/9585 (0.05)	41,202	0.12	−0.08 (−0.22–0.07)	0.60 (0.24–1.52)	0.280	0.51 (0.25–1.01)	0.054
Control	39/38,340 (0.10)	197,786	0.20		1		1	
Urban resident								
CKD	7/7238 (0.10)	32,743	0.21	−0.08 (−0.27–0.12)	0.74 (0.33–1.63)	0.450	0.65 (0.34–1.22)	0.175
Control	45/28,952 (0.16)	156,390	0.29		1		1	
Rural resident								
CKD	2/9641 (0.02)	39,133	0.05	−0.13 (−0.26–0.01)	0.27 (0.07–1.14)	0.075	0.33 (0.14–0.81)	0.015 *
Control	34/38,564 (0.09)	193,745	0.18		1		1	

Abbreviation: CKD, chronic kidney disease; IR, incidence rate; IRD, incidence rate difference; PY, person-year. * Significance at *p* < 0.05. ^†^ Adjusted for age, sex, income, region of residence, obesity, smoking, alcohol consumption, SBP, DBP, fasting blood glucose, total cholesterol, and CCI scores.

## Data Availability

All data are available from the database of National Health Insurance Sharing Service (NHISS) https://nhiss.nhis.or.kr/ (accessed on 1 March 2024). NHISS allows access to all of these data for any researcher who promises to follow the research ethics at some processing charge. If you want to access the data of this article, you can download them from the website after promising to follow the research ethics.

## Supplementary Materials

The following supporting information can be downloaded at: https://www.mdpi.com/article/10.3390/microorganisms12122614/s1, Table S1: Subgroup analyses of crude and overlap propensity score weighted hazard ratios (95% confidence interval) of CKD for deep neck infection; Table S2: Subgroup analyses of crude and overlap propensity score weighted hazard ratios (95% confidence interval) of CKD for peritonsillar abscess.
