# Supplementary material for: Chronic Kidney Disease and Infection Risk: A Lower Incidence of Peritonsillar Abscesses in Specific CKD Subgroups in a 16-Year Korean Nationwide Cohort Study
Source: Microorganisms. 2024 Dec 18;12(12):2614. doi: 10.3390/microorganisms12122614 (PMC11679683; doi:10.3390/microorganisms12122614)
Supplement: Supplementary file 1 [file microorganisms-12-02614-s001.zip › microorganisms-3331802-supplementary.pdf]

Table S1. Subgroup analyses of crude and overlap propensity score weighted hazard ratios (95% confidence interval) of CKD for deep neck infection

	N of event / N of total (%)	Follow-up duration (PY)	IR per 1000 (PY)	IRD (95% CI)	Hazard ratios for deep neck infection			
					Crude	P-value	Overlap weighted model †	P-value
Underweight								
CKD	0 / 446 (0.00)	1,368	0.00	-0.20 (-0.94 to 0.55)	N/A	0.997	N/A	0.999
Control	2 / 2,320 (0.09)	10,243	0.20		1		1	
Normal weight								
CKD	3 / 5,173 (0.06)	21,269	0.14	0.12 (0.02 to 0.21)	5.43 (1.10 to 27.0)	0.038*	4.82 (0.91 to 25.6)	0.065
Control	3 / 24,073 (0.01)	124,249	0.02		1		1	
Overweight								
CKD	0 / 4,428 (0.00)	19,908	0.00	-0.01 (-0.05 to 0.03)	N/A	0.997	N/A	1
Control	1 / 18,259 (0.01)	96,848	0.01		1		1	
Obese								
CKD	1 / 6,832 (0.01)	29,366	0.03	-0.02 (-0.10 to 0.07)	0.67 (0.08 to 5.57)	0.711	0.81 (0.18 to 3.66)	0.783
Control	6 / 22,864 (0.03)	119,156	0.05		1		1	
Non-smoker								
CKD	3 / 10,793 (0.03)	47,642	0.06	0.02 (-0.05 to 0.09)	1.39 (0.38 to 5.06)	0.615	1.37 (0.47 to 4.01)	0.564
Control	10 / 43,640 (0.02)	230,277	0.04		1		1	
Past and current smoker								
CKD	1 / 6,086 (0.02)	24,269	0.04	0.02 (-0.04 to 0.09)	2.42 (0.22 to 26.8)	0.47	3.04 (0.33 to 27.8)	0.326
Control	2 / 23,876 (0.01)	120,219	0.02		1		1	
Alcohol consumption <1 time a week								
CKD	3 / 12,274 (0.02)	53,267	0.06	0.02 (-0.05 to 0.08)	1.20 (0.33 to 4.29)	0.784	1.33 (0.45 to 3.95)	0.604
Control	11 / 46,881 (0.02)	245,237	0.04		1		1	
Alcohol consumption ≥1 time a week								
CKD	1 / 4,605 (0.02)	18,644	0.05	0.04 (-0.02 to 0.11)	5.91 (0.37 to 95.1)	0.21	4.03 (0.47 to 34.8)	0.206
Control	1 / 20,635 (0.00)	105,259	0.01		1		1	
SBP < 140 mmHg and DBP < 90 mmHg								

CKD	2 / 11,069 (0.02)	45,381	0.04	0.01 (-0.05 to 0.07)	1.34 (0.28 to 6.32)	0.711	1.41 (0.40 to 4.98)	0.595
Control	8 / 49,142 (0.02)	247,596	0.03		1		1	
SBP ≥ 140 mmHg or DBP ≥ 90 mmHg								
CKD	2 / 5,810 (0.03)	26,530	0.08	0.04 (-0.06 to 0.13)	1.78 (0.33 to 9.74)	0.505	2.16 (0.48 to 9.82)	0.319
Control	4 / 18,374 (0.02)	102,900	0.04		1		1	
Fasting blood glucose < 100 mg/dL								
CKD	3 / 7,882 (0.04)	36,372	0.08	0.04 (-0.03 to 0.12)	2.10 (0.56 to 7.93)	0.272	2.14 (0.71 to 6.40)	0.175
Control	8 / 37,512 (0.02)	210,479	0.04		1		1	
Fasting blood glucose ≥ 100 mg/dL								
CKD	1 / 8,997 (0.01)	35,539	0.03	0.00 (-0.06 to 0.06)	0.95 (0.11 to 8.55)	0.966	0.89 (0.12 to 6.46)	0.909
Control	4 / 30,004 (0.01)	140,017	0.03		1		1	
Total cholesterol < 200 mg/dL								
CKD	1 / 10,463 (0.01)	41,344	0.02	-0.01 (-0.06 to 0.05)	0.75 (0.09 to 6.21)	0.787	0.70 (0.12 to 3.96)	0.687
Control	6 / 39,657 (0.02)	194,503	0.03		1		1	
Total cholesterol ≥ 200 mg/dL								
CKD	3 / 6,416 (0.05)	30,567	0.10	0.06 (-0.03 to 0.14)	2.47 (0.62 to 9.89)	0.201	2.93 (0.88 to 9.71)	0.078
Control	3 / 6,416 (0.05)	30,567	0.10		1		1	
CCI scores = 0								
CKD	2 / 4,265 (0.05)	19,651	0.10	0.07 (-0.01 to 0.16)	3.84 (0.75 to 19.8)	0.108	3.96 (1.00 to 15.8)	0.051
Control	5 / 36,096 (0.01)	193,236	0.03		1		1	
CCI scores = 1								
CKD	0 / 2,144 (0.00)	8,394	0.00	-0.05 (-0.19 to 0.10)	N/A	0.996	N/A	0.998
Control	3 / 12,622 (0.02)	64,936	0.05		1		1	
CCI scores ≥ 2								
CKD	2 / 10,470 (0.02)	43,866	0.05	0.01 (-0.07 to 0.08)	1.02 (0.19 to 5.55)	0.986	1.34 (0.27 to 6.57)	0.722
Control	4 / 18,798 (0.02)	92,324	0.04		1		1	

Abbreviation: CKD, Chronic kidney disease; IR, incidence rate; IRD, incidence rate difference; PY, person-year.

* Significance at $P < 0.05$. † Adjusted for age, sex, income, region of residence, obesity, smoking, alcohol consumption, SBP, DBP, fasting blood glucose, total cholesterol, and CCI scores.

Table S2. Subgroup analyses of crude and overlap propensity score weighted hazard ratios (95% confidence interval) of CKD for peritonsillar abscess

	N of event / N of total (%)	Follow-up duration (PY)	IR per 1000 (PY)	IRD (95% CI)	Hazard ratios for peritonsillar abscess			
					Crude	P-value	Overlap weighted model †	P-value
Underweight								
CKD	0 / 446 (0.00)	1368	0.00	-0.10 (-0.62 to 0.43)	N/A	0.998	N/A	1.000
Control	1 / 2320 (0.04)	10,250	0.10		1		1	
Normal weight								
CKD	2 / 5173 (0.04)	21,268	0.09	-0.14 (-0.35 to 0.07)	0.39 (0.09 to 1.62)	0.194	0.50 (0.20 to 1.23)	0.131
Control	29 / 24,073 (0.12)	124,110	0.23		1		1	
Overweight								
CKD	2 / 4428 (0.05)	19,897	0.10	-0.15 (-0.38 to 0.08)	0.40 (0.09 to 1.69)	0.211	0.31 (0.10 to 0.91)	0.034*
Control	24 / 18,259 (0.13)	96,719	0.25		1		1	
Obese								
CKD	5 / 6832 (0.07)	29,343	0.17	-0.04 (-0.22 to 0.14)	0.78 (0.30 to 2.05)	0.621	0.67 (0.31 to 1.47)	0.320
Control	25 / 22,864 (0.11)	119,056	0.21		1		1	
Non-smoker								
CKD	3 / 10,793 (0.03)	47,642	0.06	-0.13 (-0.25 to 0.00)	0.33 (0.10 to 1.07)	0.064	0.22 (0.09 to 0.55)	0.001*
Control	43 / 43,640 (0.10)	230,073	0.19		1		1	
Past and current smoker								
CKD	6 / 6086 (0.10)	24,234	0.25	-0.05 (-0.29-0.18)	0.79 (0.33-1.87)	0.586	0.90 (0.46-1.74)	0.752
Control	36 / 23,876 (0.15)	120,062	0.30		1		1	
Alcohol consumption <1 time a week								
CKD	7 / 12,274 (0.06)	53,242	0.13	-0.08 (-0.21-0.05)	0.61 (0.28-1.33)	0.213	0.55 (0.31-0.99)	0.046*
Control	52 / 46,881 (0.11)	245,013	0.21		1		1	
Alcohol consumption ≥1 time a week								
CKD	2 / 4605 (0.04)	18,634	0.11	-0.15 (-0.39-0.09)	0.40 (0.09-1.68)	0.210	0.33 (0.11-1.00)	0.050*
Control	27 / 20,635 (0.13)	105,122	0.26		1		1	
SBP < 140 mmHg and DBP < 90 mmHg								

CKD	6 / 11,069 (0.05)	45,354	0.13	-0.10 (-0.24-0.05)	0.56 (0.24-1.31)	0.184	0.50 (0.27-0.90)	0.021*
Control	56 / 49,142 (0.11)	247,334	0.23		1		1	
SBP ≥ 140 mmHg or DBP ≥ 90 mmHg								
CKD	3 / 5810 (0.05)	26,522	0.11	-0.11 (-0.30-0.08)	0.49 (0.15-1.65)	0.252	0.53 (0.20-1.44)	0.217
Control	23 / 18,374 (0.13)	102,801	0.22		1		1	
Fasting blood glucose < 100 mg/dL								
CKD	2 / 7882 (0.03)	36,362	0.06	-0.16 (-0.33--0.01)	0.24 (0.06-0.98)	0.047*	0.23 (0.10-0.55)	<0.001*
Control	47 / 37,512 (0.13)	210,279	0.22		1		1	
Fasting blood glucose ≥ 100 mg/dL								
CKD	7 / 8997 (0.08)	35,514	0.20	-0.03 (-0.21 to 0.14)	0.85 (0.37 to 1.92)	0.690	0.90 (0.45 to 1.81)	0.770
Control	32 / 30,004 (0.11)	139,856	0.23		1		1	
Total cholesterol < 200 mg/dL								
CKD	4 / 10,463 (0.04)	41,330	0.10	-0.12 (-0.27 to 0.03)	0.42 (0.15 to 1.18)	0.099	0.31 (0.14 to 0.69)	0.004*
Control	43 / 39,657 (0.11)	194,304	0.22		1		1	
Total cholesterol ≥ 200 mg/dL								
CKD	5 / 6416 (0.08)	30,546	0.16	-0.07 (-0.25 to 0.11)	0.70 (0.27 to 1.77)	0.448	0.75 (0.38 to 1.47)	0.406
Control	36 / 27,859 (0.13)	155,831	0.23		1		1	
CCI scores = 0								
CKD	5 / 4265 (0.12)	19,630	0.25	0.04 (-0.17 to 0.26)	1.18 (0.47 to 2.99)	0.727	1.18 (0.66 to 2.11)	0.578
Control	41 / 36,096 (0.11)	193,063	0.21		1		1	
CCI scores = 1								
CKD	0 / 2144 (0.00)	8394	0.00	-0.22 (-0.53 to 0.10)	N/A	0.992	N/A	0.993
Control	14 / 12,622 (0.11)	64,867	0.22		1		1	
CCI scores ≥ 2								
CKD	4 / 10,470 (0.04)	43,852	0.09	-0.17 (-0.33 to -0.01)	0.34 (0.12 to 0.98)	0.046*	0.25 (0.09 to 0.73)	0.011*
Control	24 / 18,798 (0.13)	92,205	0.26		1		1	

Abbreviation: CKD, Chronic kidney disease; IR, incidence rate; IRD, incidence rate difference; PY, person-year.
* Significance at P < 0.05. † Adjusted for age, sex, income, region of residence, obesity, smoking, alcohol consumption, SBP, DBP, fasting blood glucose, total cholesterol, and CCI scores.
